# Cardiovascular risk factors are associated with augmented thrombogenicity in healthy individuals: analysis using the Total Thrombus-formation Analysis System

**DOI:** 10.1186/s12959-021-00341-3

**Published:** 2021-11-17

**Authors:** Yuu Oda, Takashi Ito, Yoichiro Yamada, Tadashi Koga, Tomoka Nagasato, Tomoko Ohnishi-Wada, Kazuya Hosokawa, Hiroyuki Fukase, Teruto Hashiguchi, Ikuro Maruyama

**Affiliations:** 1 Department of Systems Biology in Thromboregulation, Kagoshima University Graduate School of Medical and Dental Sciences, Kagoshima, Japan; 2 Department of Biomedical Laboratory Sciences, Faculty of Life Sciences, Kumamoto University, 4-24-1 Kuhonji, Kumamoto, 862-0976 Japan; 3 PPD-SNBL K.K., Kagoshima, Japan; 4 Clinical Study Support, Inc., Nagoya, Japan; 5 Research Institute, Fujimori Kogyo Co., Ltd., Yokohama, Japan; 6 Clinical Research Hospital, Tokyo, Japan; 7 Department of Laboratory and Vascular Medicine, Kagoshima University Graduate School of Medical and Dental Sciences, Kagoshima, Japan

## Abstract

**Background:**

Rupture of an atherosclerotic plaque and subsequent exposure of the subendothelial prothrombotic matrix to blood cause arterial thrombosis. Circulating platelets play an indispensable role in the growth of arterial thrombi partially owing to their unique ability to adhere to the subendothelial matrix and to aggregate to each other under flow conditions. Recently, the Total Thrombus-formation Analysis System (T-TAS) was developed for ex vivo analysis of the thrombogenic potential of whole blood samples under flow conditions. Despite the potential clinical utility of the T-TAS in assessing the risk for thrombosis and bleeding, reference intervals for T-TAS analysis in healthy individuals have not been determined.

**Methods:**

In total, 122 whole blood samples were collected from healthy volunteers ranging in age from 25 to 45 years. T-TAS analysis and hematological, physiological, and lifestyle assessments were conducted in these subjects. Whole blood samples anticoagulated with hirudin were perfused into a collagen-coated microchip (PL chip). The time to 10 kPa and the area under the flow pressure curve up to 10 min (AUC_10_) were analyzed as representative variables for thrombogenic potential. Reference intervals, which were defined as 2.5–97.5 percentiles, were determined. Additionally, univariate and multivariate analyses were performed to identify factors associated with the AUC_10_ in the T-TAS.

**Results:**

The time to 10 kPa and the AUC_10_ widely varied, even in healthy volunteers. The reference intervals were 1.50–4.02 min and 223.4–456.8, respectively, at a shear rate of 1500 s^− 1^. Univariate and multivariate analyses showed that platelet counts were most significantly associated with the AUC_10_ of the T-TAS. The presence of one or more cardiovascular risk factors of a high body mass index, a high pulse pressure, high fasting serum glucose levels, high low-density lipoprotein-cholesterol levels, a history of smoking, and no habitual exercise, had the second largest effect on the AUC_10_ of the T-TAS.

**Conclusions:**

Healthy volunteers who had any cardiovascular risk factors showed augmented thrombogenicity, even in artificial uniform capillaries, compared with those without any risk factors in the T-TAS.

**Supplementary Information:**

The online version contains supplementary material available at 10.1186/s12959-021-00341-3.

## Background

Arterial thrombosis is a major healthcare problem not only in industrialized countries, but also in low- and middle-income countries [1]. The development of occlusive thrombi in coronary or cerebral arteries results in acute myocardial infarction or ischemic stroke, respectively, and is currently the most common cause of morbidity and mortality globally. The essential triggers for arterial thrombosis include rupture of an atherosclerotic plaque and subsequent exposure of the subendothelial prothrombotic matrix [2]. Circulating platelets play an indispensable role in the growth of arterial thrombi in part owing to their unique ability to adhere to the subendothelial matrix and to aggregate to each other under rapid flow conditions [3].

The Total Thrombus-formation Analysis System (T-TAS®, Fujimori Kogyo Co., Ltd., Yokohama, Japan) was developed for ex vivo analysis of the thrombogenic potential of whole blood under rapid flow conditions [4]. In this system, whole blood samples are perfused into artificial capillaries coated with collagen, a major subendothelial matrix protein. The thrombogenic potential is then quantified by measuring an elevation in internal flow pressure caused by growth of obstructive thrombi. Recent studies have shown that this system may be useful in evaluating the therapeutic efficacy of antiplatelet drugs [5, 6], risk of bleeding in patients undergoing percutaneous coronary intervention [7], and risk of thrombotic complications in hypoglycemic patients [8].

Despite the potential clinical utility, reference intervals for T-TAS analysis in healthy individuals have not been determined. Furthermore, blood components, other than platelets, contributing to the growth of obstructive thrombi in T-TAS have not been well defined. Therefore, this study aimed to collect whole blood samples from a sufficient number of qualified reference individuals to yield a minimum of 120 samples for establishing reference intervals for T-TAS analysis [9, 10]. This study shows that the thrombogenic potential widely varies among healthy individuals and that cardiovascular risk factors are associated with augmented thrombogenicity, even in artificial uniform capillaries.

## Methods

### Study subjects

This study was conducted in accordance with the Declaration of Helsinki. This study was approved by the Institutional Review Boards of CPC Clinical Trial Hospital and Kagoshima University Graduate School of Medical and Dental Sciences, both in Kagoshima, Japan. Written informed consent was obtained from all subjects prior to their participation in the study. Healthy volunteers ranging in age from 25 to 45 years were recruited in CPC Clinical Trial Hospital. Subjects participating in other clinical trials or receiving drugs and/or nutritional supplements were excluded. One hundred twenty-five volunteers were enrolled, among whom 122 (88 men and 34 women) were eligible for further analysis as healthy individuals. Daily habits of smoking, exercise, drinking, and eating were assessed by self-report questionnaires. Height, weight, blood pressure, and pulse rate were assessed in all healthy volunteers. None of them had a bleeding tendency.

### Hematological examination

Blood was collected from the forearm using a 21-G needle and vacuum blood collection tubes after fasting for at least 6 h. Blood samples were anticoagulated with hirudin (Roche Diagnostics GmbH, Mannheim, Germany), ethylenediaminetetraacetic acid, or sodium citrate, which were used for T-TAS assays, complete blood cell counts, or coagulation assays, respectively. Serum samples were obtained from blood without anticoagulation, and were used for measuring levels of bilirubin, aspartate aminotransferase, alanine aminotransferase, lactate dehydrogenase, γ-glutamyl transpeptidase, albumin, globulin, creatine phosphokinase, amylase, glucose, low-density lipoprotein (LDL)-cholesterol, high-density lipoprotein (HDL)-cholesterol, triglycerides, urea nitrogen, creatinine, uric acid, sodium, potassium, and chloride.

### Analysis of ex vivo thrombogenic potential using the T-TAS

The process of platelet thrombus formation was evaluated using the T-TAS, as described previously [4, 5]. Briefly, whole blood specimens (350 μL) anticoagulated with hirudin were perfused into a collagen-coated microchip (PL chip, Fujimori Kogyo Co., Ltd.) at a constant flow rate of 12, 18, or 24 μL/min, which corresponded to an initial wall shear rate of 1000, 1500, or 2000 s^− 1^. The growth of thrombus inside the microchip was quantitatively evaluated by monitoring flow pressure over time. The time to 10 kPa and the area under the flow pressure curve up to 10 min (AUC_10_) were analyzed as representative variables for the thrombogenic potential.

### Definition of cardiovascular risk factors in this study

A body mass index (BMI) ≥ 25 kg/m^2^, a pulse pressure ≥ 60 mmHg, fasting serum glucose levels ≥110 mg/dL, LDL-cholesterol levels ≥120 mg/dL, a history of smoking, and no habitual exercise were defined as cardiovascular risk factors, according to previous studies [11, 12].

### Statistical analysis

Statistical analyses were performed using IBM SPSS v26 (IBM Corp., Armonk NY, USA) and GraphPad Prism v8 (GraphPad Software, San Diego, CA, USA). Data are presented as the median (lower quartile−upper quartile), means ± standard deviations, or 2.5–97.5 percentiles. The relationships between the AUC_10_ (at a shear rate of 1500 s^− 1^) in the T-TAS and other explanatory variables were analyzed by Spearman’s rank correlation test, the Mann–Whitney U test, or multiple regression analysis with the stepwise method. A *P* value of < 0.05 was considered significant.

## Results

**Fig. 1:**
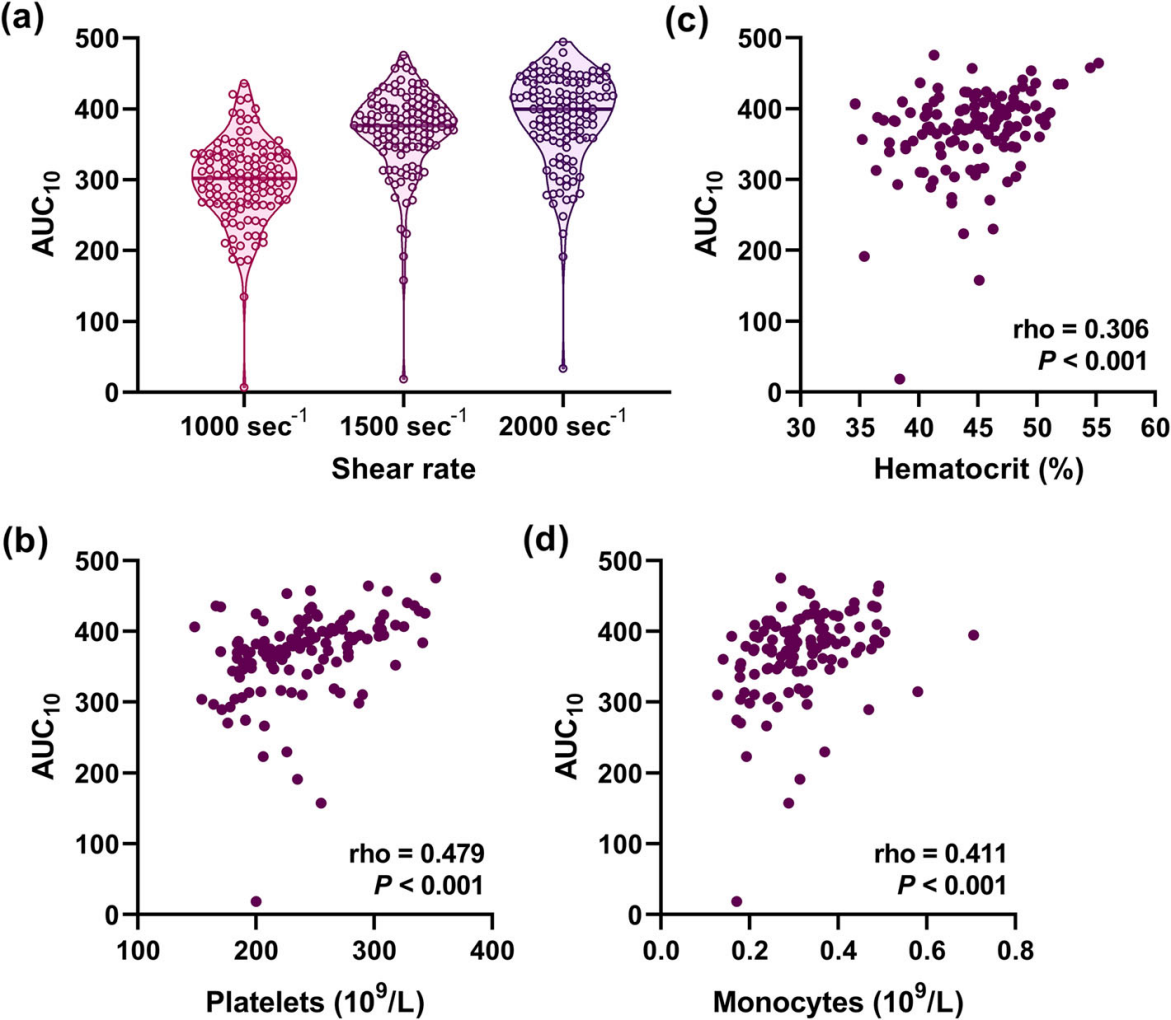
Quantitative analysis of the thrombogenic potential in 122 healthy individuals. (a) The area under the flow pressure curve in T-TAS PL chips up to 10 min (AUC_10_) was assessed as a representative variable for thrombogenic potential. The initial wall shear rates were set at 1000, 1500, or 2000 s^− 1^. The distribution of the AUC_10_ in 122 healthy volunteers is shown as violin plots. Bold horizontal lines indicate median values. (b–d) Correlations between the AUC_10_ (at a shear rate of 1500 s^− 1^) in the T-TAS and platelet counts (b), hematocrit (c), and monocyte counts (d) were analyzed by Spearman’s rank correlation test

To establish reference intervals for the T-TAS, we analyzed 122 whole blood samples collected from healthy volunteers. The AUC_10_ widely varied, even in healthy volunteers (Fig. 1a). The reference interval, which was defined as 2.5–97.5 percentiles, was 223.4–456.8 at the shear rates of 1500 s^− 1^ (Table 1). The AUC_10_ increased as the setting of the shear rate in the assay increased, and the reference intervals were 186.6–411.9 and 248.0–465.6 at the shear rates of 1000 and 2000 s^− 1^, respectively.

**Table 1:** Reference intervals of T-TAS values based on data from 122 healthy individuals

Shear rate	Measurement	Mean	Median	Reference interval (95%)		
1000 s^− 1^	T10 (min:s)	03:02	02:48	01:41	–	04:39
	AUC_10_	298.0	301.9	186.6	–	411.9
1500 s^−1^	T10 (min:s)	02:34	02:25	01:30	–	04:01
	AUC_10_	367.6	376.6	223.4	–	456.8
2000 s^−1^	T10 (min:s)	02:35	02:22	01:33	–	04:02
	AUC_10_	385.1	399.8	248.0	–	465.6

T10: time to 10 kPa

We then analyzed what determined the heterogeneity of the AUC_10_ in the T-TAS. Among 50 variables in hematological, physiological, and lifestyle parameters (Supplementary Table 1), platelet counts showed the most significant association (rho = 0.479, *P* <  0.001) with the AUC_10_ of the T-TAS at the shear rates of 1500 s^− 1^ (Fig. 1b). Additionally, the hematocrit and the number of leukocytes, especially that of a monocyte fraction, showed significant associations with the AUC_10_ of the T-TAS (Fig. 1c, d). In more detailed analysis, the hematocrit and leukocyte counts were associated with the initial rise in flow pressure and platelet counts were associated with the following phase of thrombus growth (data not shown).

**Fig. 2:**
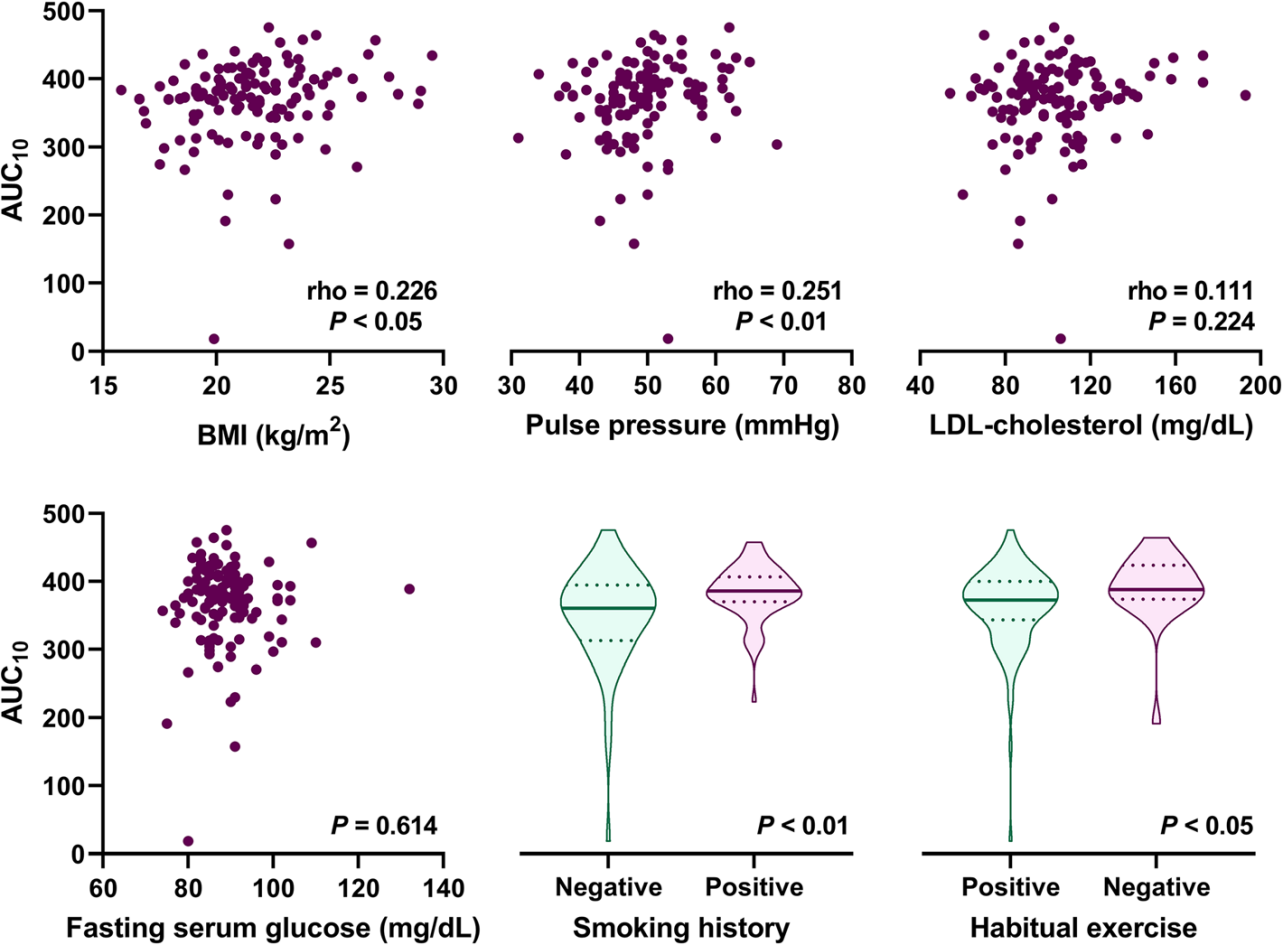
Correlations between the thrombogenic potential and cardiovascular risk factors in 122 healthy individuals. Correlations between the AUC_10_ (at a shear rate of 1500 s^− 1^) in the T-TAS and BMI, pulse pressure, LDL-cholesterol levels, fasting serum glucose levels, smoking history, and habitual exercise were analyzed by Spearman’s rank correlation test or the Mann–Whitney U test

**Fig. 3:**
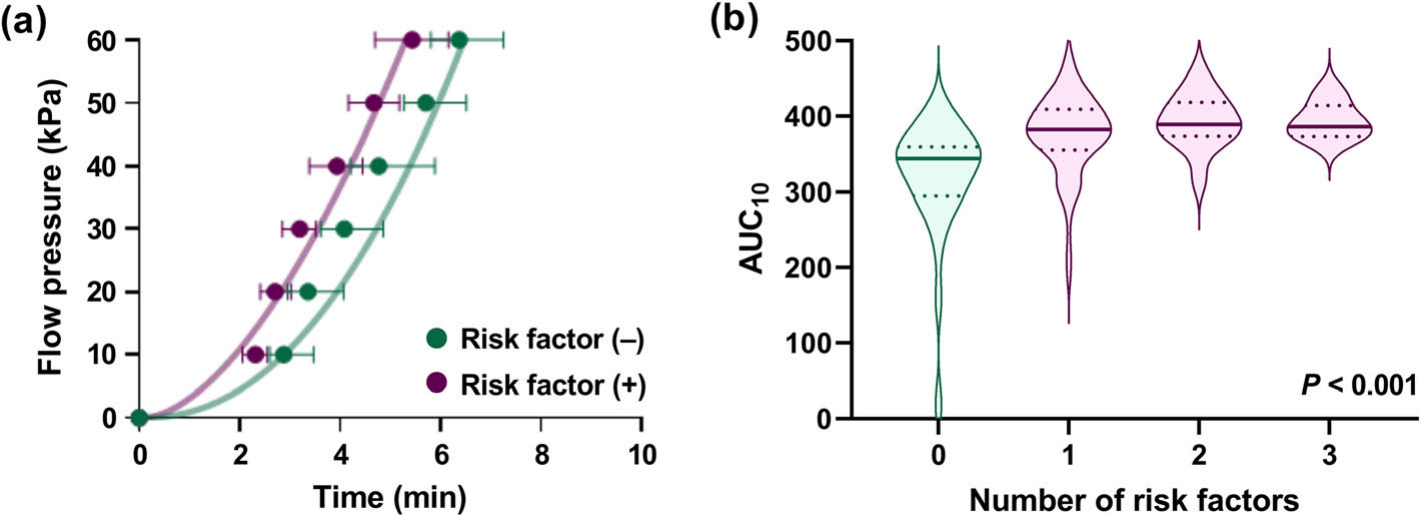
Healthy individuals with one or more cardiovascular risk factors show augmented thrombogenicity. (a) The times to 10, 20, 30, 40, 50, and 60 kPa (at a shear rate of 1500 s^− 1^) in the T-TAS in healthy individuals with or without cardiovascular risk factor(s) are shown. Filled circles and error bars indicate medians and interquartile ranges, respectively. A nonlinear curve fit model was used for estimating theoretical flow pressure curves in individuals with or without cardiovascular risk factor(s). (b) The distribution of the AUC_10_ in the T-TAS in healthy individuals without (*n* = 28) or with one (*n* = 59), two (*n* = 26), or three (*n* = 9) cardiovascular risk factors is shown as violin plots. Bold and dotted horizontal lines indicate medians and quartiles, respectively. The correlation between the AUC_10_ (at a shear rate of 1500 s^− 1^) in the T-TAS and the number of cardiovascular risk factors was analyzed by Spearman’s rank correlation test

We then analyzed factors, other than blood cells, that might be associated with augmented thrombogenicity in the T-TAS. Serum potassium levels were unexpectedly correlated with the AUC_10_ of the T-TAS (Table 2). Among cardiovascular risk factors, BMI and pulse pressure were weakly, but significantly, associated with the AUC_10_ of the T-TAS (Fig. 2). LDL-cholesterol and fasting serum glucose levels were not associated with the AUC_10_ of the T-TAS. A positive smoking history and negative habitual exercise were associated with a higher AUC_10_ of the T-TAS. Subjects who had any of the above-mentioned cardiovascular risk factors showed augmented thrombogenicity in the T-TAS compared with those without any risk factors (Fig. 3a). The number of risks was significantly associated with the AUC_10_ of the T-TAS (Fig. 3b).

**Table 2:** Univariate and multivariate analyses for identifying factors associated with the AUC_10_ of the T-TAS

Explanatory variable	Correlation analysis		Multivariate analysis		
	Spearman’s rho	Significance probability	Standardization coefficient	t value	Significance probability
Platelets	0.479	< 0.001	0.319	4.30	< 0.001
Risk factor(s)	0.459	< 0.001	0.241	3.17	0.002
Monocytes	0.411	< 0.001	0.201	2.62	0.010
Potassium	0.322	< 0.001	0.225	3.02	0.003
Hematocrit	0.306	0.001	0.204	2.61	0.010

We then performed multivariate analysis to estimate the degree of the contribution of each factor to thrombogenicity in the T-TAS. Multiple regression analysis showed five main factors that had a significant effect on thrombogenicity in the T-TAS (Table 2). These factors were platelet counts (t = 4.30, *P* <  0.001), the presence of one or more cardiovascular risk factors (t = 3.17, *P* = 0.002), serum potassium levels (t = 3.02, *P* = 0.003), monocyte counts (t = 2.62, *P* = 0.010), and the hematocrit (t = 2.61, *P* = 0.010), which collectively accounted for as much as 40.6% of the variance in thrombogenicity in the T-TAS. The presence of cardiovascular risk factors had the second largest effect on thrombogenicity in the T-TAS following platelet counts.

## Discussion

In this study, we analyzed 122 whole blood samples collected from healthy volunteers and clarified reference intervals for the T-TAS. Substantial heterogeneity was observed even in healthy individuals, and some of them showed unexpectedly low AUC_10_ values. The low AUC_10_ values were unlikely due to random technical errors because the AUC_10_ values were consistently low in the same individual in the different experimental conditions (three different flow rates with different microchips). Healthy subjects with low AUC_10_ values (less than 200) were also found in previous reports [13]. Although they had no bleeding tendency, few, if any, abnormalities in other clinical chemistry tests, or no drugs interfering the process of thrombus formation, they may pose a potential risk for bleeding events. Longitudinal follow-up studies will be required.

Reference intervals defined in this study were consistent with a previous report, where 31 healthy subjects (11 men and 20 women) were examined [13]. However, AUC_10_ values were slightly higher in our study. This may be due to a different composition of participants in terms of their age and gender. Participants in our study include a larger number of young men, who typically show higher AUC_10_ values. Gender-specific and age-specific reference intervals may be required in the future.

In this study, we identified factors for determining the heterogeneity of thrombogenicity in the T-TAS. These factors included the platelet number, the leukocyte number, the hematocrit, serum potassium levels, and the presence of one or more cardiovascular risk factors. Although a small portion of healthy subjects in this study showed slightly elevated serum glucose, serum triglyceride (and/or low HDL-cholesterol), or blood pressure levels above the criteria for metabolic syndrome [14], they were considered to be otherwise healthy.

In this study, platelet counts showed the strongest correlation with the AUC_10_ at the shear velocity of 1500 s^− 1^. Platelet counts were more clearly associated with the time to occlusion (60 kPa) than with the time to 10 kPa. Therefore, the number of platelets might be more important in the later stage of the thrombus growth process than in the earlier stage of platelet adhesion. We previously reported that aspirin prolonged the time to 60 kPa, but not the time to 10 kPa [5]. This finding suggested that not only platelet counts but also platelet activity may play an important role in the later stage of thrombus growth.

Leukocytes are also involved in the thrombus formation process [15]. They are larger than red blood cells or platelets, and therefore, can decrease the rate of blood flow, enabling easier growth of a thrombus under flow conditions. Among leukocytes, monocytes showed the strongest correlation with thrombogenicity in the T-TAS. This finding may have occurred because monocytes and/or platelet–monocyte complexes can promote the growth of platelet-rich thrombi [16, 17] or simply because monocytes have the largest nucleus, which can generate resistance to blood flow in capillaries.

The hematocrit was more clearly associated with the time to 10 kPa than with the time to 60 kPa in this study. This finding suggested that the volume of red blood cells played a role in the initial stage of thrombus growth. Previous studies have shown experimentally and clinically that red blood cells determine blood viscosity [18, 19]. Furthermore, under blood flow conditions, red blood cells generate an axial stream, which secondarily generates near-wall excess of platelets and plasma proteins. Therefore, an increase in the hematocrit may lead to a higher platelet density around the capillary wall, which enables platelets to more readily attach to the wall [20, 21].

Serum potassium levels varied within a relatively narrow range (3.6–4.7 mEq/L) in this study. However, a significant correlation was observed between serum potassium levels and thrombogenicity in the T-TAS. There were no correlations of potassium levels with other parameters, except for a weak correlation with a BMI > 25 kg/m^2^. Potassium may directly affect thrombus formation without any effect on coagulation and fibrinolysis [22]. Potassium may also be an “innocent bystander” in hemolysis, which can be a confounding factor directly related to thrombus formation. Hemolysis results in the extracellular release of adenosine diphosphate, calcium ions, and hemoglobin, all of which activate platelets [23, 24]. Further studies are required to examine the effect of potassium and/or hemolysis on thrombus formation in the T-TAS, although completely excluding minor hemolysis during the blood sampling is difficult.

In this study, a BMI ≥ 25 kg/m2, a pulse pressure ≥ 60 mmHg, fasting blood glucose levels ≥110 mg/dL, LDL-cholesterol levels ≥120 mg/dL, a history of smoking, and a low daily physical activity level were considered as cardiovascular risk factors [25–27]. Age is another important risk factor related to cardiovascular events. However, in the present study, we did not include age as a risk factor because all of the subjects were young (25–45 years). Among cardiovascular risk factors analyzed in the present study, a history of smoking showed the strongest correlation with thrombus formation. Smoking may augment thrombogenicity by enhancing platelet agglutinability via oxidative stress or by increasing blood viscosity due to secondary polycythemia [28]. Systolic blood pressure and pulse pressure were weakly correlated with thrombus formation in the T-TAS, with a slightly stronger correlation observed in pulse pressure. In previous studies in older people, pulse pressure was a risk factor for cardiovascular events and was independent of systolic blood pressure and average blood pressure [29, 30]. In this study, although the majority of subjects were young, a correlation between pulse pressure and thrombus formation was observed.

Generally, cardiovascular risk factors are considered to increase the incidence of ischemic or hemorrhagic vascular events by accelerating atherosclerosis or vascular injury [31, 32]. However, the effect of cardiovascular risk factors on the thrombogenicity of blood components rather than vascular components has rarely been discussed. In this study, we showed that the presence of cardiovascular risk factors was associated with augmented thrombogenicity, even in the artificial uniform capillaries, in the T-TAS. This finding suggests that cardiovascular risk factors have a significant effect on the thrombogenicity of blood components.

Longitudinal follow-up studies are required in the future. Determining whether individuals who show a high AUC_10_ in the T-TAS will experience thrombotic events more frequently than those who show a low AUC_10_ is important. Determining whether individuals with a very low AUC_10_ in the T-TAS will experience bleeding events is also important. Findings from these investigations will provide confirmatory evidence and the basis of a strategy for assessing and managing cardiovascular risks.

## Conclusions

Reference intervals for T-TAS analysis were determined by an analysis of 122 whole blood samples collected from healthy individuals. The platelet number, the leukocyte number, the hematocrit, serum potassium levels, and the presence of one or more cardiovascular risk factors determine the heterogeneity of the thrombogenic potential in the T-TAS

## Supplementary Information

**Additional file 1.**

## Data Availability

The datasets used and/or analyzed during the current study are available from the corresponding author on reasonable request.

## Abbreviations

AUC_10_: Area under the flow pressure curve up to 10 min
BMI: Body mass index
HDL: High-density lipoprotein
LDL: Low-density lipoprotein
SD: Standard deviation
T-TAS: Total Thrombus-formation Analysis System
