# Supplementary material for: Cardiovascular risk factors are associated with augmented thrombogenicity in healthy individuals: analysis using the Total Thrombus-formation Analysis System
Source: Thromb J. 2021 Nov 17;19:88. doi: 10.1186/s12959-021-00341-3 (PMC8597268; doi:10.1186/s12959-021-00341-3)
Supplement: Supplementary file 1 — Additional file 1. [file 12959_2021_341_MOESM1_ESM.pdf]

Supplementary Table 1. Physiological, hematological, and lifestyle parameters of the study subjects.

Physiological and hematological parameters	Mean ± SD	Minimum	Maximum	Spearman's rho
Age (years)	28.55 ± 6.51	20	45	-.040
Body mass index (kg/m^2)	21.74 ± 2.66	15.8	29.5	.217
Systolic blood pressure (mmHg)	113.76 ± 8.35	92	132	.185
Diastolic blood pressure (mmHg)	64.09 ± 6.93	42	82	-.004
Pulse pressure (mmHg)	49.67 ± 6.675	31	69	.251
Pulse rate (bpm)	61.70 ± 8.43	42	87	.099
Total bilirubin (mg/dL)	0.93 ± 0.35	0.38	2.37	-.081
Direct bilirubin (mg/dL)	0.06 ± 0.02	0.02	0.13	.115
Alkaline phosphatase (IU/L)	172.45 ± 50.27	39	318	-.039
Aspartate aminotransferase (IU/L)	18.22 ± 4.88	10	43	.072
Alanine aminotransferase (IU/L)	18.016 ± 13.52	5	125	.233
Lactate dehydrogenase (IU/L)	154.51 ± 23.03	102	230	-.002
γ-glutamyl transpeptidase (IU/L)	23.14 ± 20.00	7	167	.136
Total protein (g/dL)	7.02 ± 0.40	6.2	8.1	.112
Albumin (g/dL)	4.68 ± 0.32	3.8	5.5	.106
Globulin (g/dL)	2.35 ± 0.35	1.6	3.6	0.049
Creatine phosphokinase (IU/L)	114.73 ± 124.32	43	1353	.038
Amylase (IU/L)	75.26 ± 24.01	28	169	.042
Glucose (mg/dL)	88.85 ± 7.61	74	132	-.046
Low-density lipoprotein cholesterol (mg/dL)	104.22 ± 23.87	54	193	.111
High-density lipoprotein cholesterol (mg/dL)	63.88 ± 13.99	35	111	-.152
Triglycerides (mg/dL)	79.43 ± 49.45	27	327	.080
Total cholesterol (mg/dL)	183.98 ± 27.66	120	270	0.048
Urea nitrogen (mg/dL)	11.86 ± 2.88	6.8	19.7	.009
Creatinine (mg/dL)	0.72 ± 0.13	0.4	0.97	.139
Uric acid (μmol/L)	5.42 ± 1.40	1.6	8.5	.118
Na (mEq/L)	139.11 ± 1.22	136	142	-.048
K (mEq/L)	4.04 ± 0.23	3.6	4.7	.322
Cl (mEq/L)	102.46 ± 2.04	99	108	-.054
White blood cells (10^3/μL)	5.77 ± 1.57	3.1	10.6	.385
Hematocrit (%)	44.40 ± 4.17	34.6	55.2	.306

Platelets (10^3/μL)	237.7 ± 4.7	148	352	.479
Lymphocytes (10^3/μL)	1.67 ± 0.41	0.74	3.08	.197
Neutrophils (10^3/μL)	3.57 ± 1.31	1.65	8.27	.365
Eosinophils (/μL)	178 ± 134	18.4	624	.065
Basophils (/μL)	36 ± 17	8.4	81.9	.165
Monocytes (/μL)	316 ± 98	128	706	.411
Prothrombin time (s)	14.54 ± 0.89	12.2	16.9	-.184
Activated partial thromboplastin time (s)	37.52 ± 4.44	27.9	55.1	.060
Fibrinogen (mg/dL)	246.99 ± 55.48	137	573	.191
D-dimer (μg/mL)	0.261 ± 0.197	0.05	1.41	-.021

Lifestyle parameters	%
Female sex	28.5
Three meals a day	61
Eating out frequently	17.1
More meat than fish	84.6
High-vegetable diet	8.9
Low-sodium diet	13
Smoking history	51.2
Drinking (≥ 5 days a week)	9.8
Regular physical activity	81.3

Mean ± standard deviation (SD), minimum, maximum, and correlation coefficient (Spearman's rho) between AUC_{10} of the T-TAS and each parameter are shown.
